# Role of different mechanisms in pro-inflammatory responses triggered by traffic-derived particulate matter in human bronchiolar epithelial cells

**DOI:** 10.1186/s12989-023-00542-w

**Published:** 2023-08-03

**Authors:** Magne Refsnes, Tonje Skuland, Rikke Jørgensen, Vegard Sæter-Grytting, Brynhild Snilsberg, Johan Øvrevik, Jørn A. Holme, Marit Låg

**Affiliations:** 1 Department of Air quality and Noise, Division of Climate and Environmental Health, Norwegian Institute of Public Health, PO Box 222, Skøyen, Oslo, 0213 Norway; 2 Department of Industrial Economics and Technology Management, Norwegian University of Science and Technology, NTNU, Trondheim, Norway; 3 Norwegian Public Roads Administration, Trondheim, Norway; 4 Department of Biosciences, Faculty of Mathematics and Natural Sciences, University of Oslo, Oslo, Norway; 5 Division of Climate and Environmental Health, Norwegian Institute of Public Health, Oslo, Norway

**Keywords:** Traffic-derived particle matter, Wear particles, Mineral particles, COX2, CYP, Heme oxygenase, HMOX2, Aryl hydrocarbon receptor, Reactive oxygen species. Non-combustion particles

## Abstract

**Background:**

Traffic-derived particles are important contributors to the adverse health effects of ambient particulate matter (PM). In Nordic countries, mineral particles from road pavement and diesel exhaust particles (DEP) are important constituents of traffic-derived PM. In the present study we compared the pro-inflammatory responses of mineral particles and DEP to PM from two road tunnels, and examined the mechanisms involved.

**Methods:**

The pro-inflammatory potential of 100 µg/mL coarse (PM_10-2.5_), fine (PM_2.5-0.18)_ and ultrafine PM (PM_0.18_) sampled in two road tunnels paved with different stone materials was assessed in human bronchial epithelial cells (HBEC3-KT), and compared to DEP and particles derived from the respective stone materials. Release of pro-inflammatory cytokines (CXCL8, IL-1α, IL-1β) was measured by ELISA, while the expression of genes related to inflammation (COX2, CXCL8, IL-1α, IL-1β, TNF-α), redox responses (HO-1) and metabolism (CYP1A1, CYP1B1, PAI-2) was determined by qPCR. The roles of the aryl hydrocarbon receptor (AhR) and reactive oxygen species (ROS) were examined by treatment with the AhR-inhibitor CH223191 and the anti-oxidant N-acetyl cysteine (NAC).

**Results:**

Road tunnel PM caused time-dependent increases in expression of CXCL8, COX2, IL-1α, IL-1β, TNF-α, COX2, PAI-2, CYP1A1, CYP1B1 and HO-1, with fine PM as more potent than coarse PM at early time-points. The stone particle samples and DEP induced lower cytokine release than all size-fractionated PM samples for one tunnel, and versus fine PM for the other tunnel. CH223191 partially reduced release and expression of IL-1α and CXCL8, and expression of COX2, for fine and coarse PM, depending on tunnel, response and time-point. Whereas expression of CYP1A1 was markedly reduced by CH223191, HO-1 expression was not affected. NAC reduced the release and expression of IL-1α and CXCL8, and COX2 expression, but augmented expression of CYP1A1 and HO-1.

**Conclusions:**

The results indicate that the pro-inflammatory responses of road tunnel PM in HBEC3-KT cells are not attributed to the mineral particles or DEP alone. The pro-inflammatory responses seem to involve AhR-dependent mechanisms, suggesting a role for organic constituents. ROS-mediated mechanisms were also involved, probably through AhR-independent pathways. DEP may be a contributor to the AhR-dependent responses, although other sources may be of importance.

**Supplementary Information:**

The online version contains supplementary material available at 10.1186/s12989-023-00542-w.

## Introduction

Air pollution is among the leading environmental risk factors for exacerbation and development of respiratory and cardiovascular disease, with particulate matter (PM) as the most important component [1–5]. Although traffic is a major source for ambient PM, its relative contribution is highly dependent on region, time-point of the study, and the PM size fraction [6]. Sources of traffic-derived particles include particles from diesel- and gasoline exhaust as well as non-exhaust particles from wear of road pavement, tires and brakes, and from road resuspension dust as a secondary source [7, 8]. The main focus of research has been on adverse health effects linked to combustion-derived particles, and in particular the pro-inflammatory effects of diesel exhaust particles (DEP) [9–11]. Inflammatory effects are considered central for respiratory diseases, including development and/or exacerbation of asthma and chronic obstructive pulmonary disease (COPD), increased occurrence of respiratory infections, and lung cancer [3, 4], as well as for cardiovascular diseases [5]. These findings have resulted in policy regulations and concurrent technological development, leading to lower emissions and lower ambient PM levels in the Western world. As a result, the physical properties and chemical composition of traffic-derived PM have been altered [8, 12]. Since more knowledge about the possible health hazard linked to this modified type of traffic PM is highly needed, there has been a substantial increase in studies of constituents from other traffic-derived sources, including various wear particles from asphalt, tires and brakes, both with respect to contribution to ambient PM levels, particle reactivity and health effects [7, 8, 13]. Due to the use of studded tires during the winter season, the relative levels of road wear particles are high in Nordic countries compared to countries with warmer climate [14]. As the size, chemical composition and reactivity of wear particles seem to be different from combustion-derived traffic particles [8], it is important to further compare their relative contribution to the toxicity and pro-inflammatory potential of size-fractioned PM samples, and to elucidate mechanisms involved in these responses.

Ambient PM is usually divided in coarse, fine and ultrafine fractions based on aerodynamic size [15]. Particles generated by wear processes are most prevalent in the coarse fraction, but also occur in the fine fraction [7]. Conversely, combustion-derived particles are mainly enriched in the PM_2.5_ and PM_0.1_ fractions [16]. Epidemiological studies indicate that short-term exposure to both coarse and fine PM is associated with respiratory hospital admissions and mortality [17, 18]. Furthermore, some animal studies [19] and in vitro experiments have reported higher pro-inflammatory responses of coarse than fine PM [19–26], whereas other animal studies [27, 28] and *in vitro studies* [29–31] show similar or higher responses for the fine than coarse PM. The relative impact of coarse versus fine and ultrafine PM depends on their specific sources, but also on the model system and health outcome examined. It is well-known that PM may cause respiratory adverse health effects through induction of local pulmonary inflammation by directly triggering release of cytokines. PM may also act by activation of respiratory neuronal reflexes or by damaging the lung epithelial barri**e**r, and contribute to increased risk for bacterial and viral infection [32]. The triggering of pro-inflammatory responses may involve various cellular signaling pathways, and the specific responses and triggering mechanisms are highly dependent on cell type. Important mechanisms include binding of specific receptors, such as the aryl hydrocarbon receptor (AhR), or disturbance of signaling pathways involving reactive oxygen species (ROS) [32–37]. More knowledge regarding these processes may help elucidating the relative contribution of particle sources to the specific cytokine responses and exacerbation and development of diseases.

We have previously demonstrated that particles derived from different stone (rock) aggregates used in road pavement may induce inflammatory responses of different magnitude [11, 38–40], suggesting that the choice of stone type may be of importance for adverse respiratory effects induced by traffic-related PM. In our recent study, particles of different sizes were sampled inside two road tunnels paved with different stone materials [29]. The results showed that fine PM (PM_2.5-0.18_) induced higher pro-inflammatory responses in human bronchial epithelial cells (HBEC3-KT cells) than coarse PM (PM_10-2.5_), whereas the potency of the ultrafine PM (< PM_0.18_) was more variable. The potency of the road tunnel PM could not be attributed to stone material in the asphalt alone [29]. Instead, the pro-inflammatory responses of the road tunnel PM were correlated to the content of organic carbon (OC) in the different PM samples on a mass basis.

The present study extends the findings of our previous study [29], and further examines the pro-inflammatory responses of some of the size-fractionated PM samples from the two road tunnels by assessing the release of pro-inflammatory cytokines and expression of genes related to inflammation, redox responses and xenobiotic metabolism. Based on the potential role of OC observed in our previous study [29], the main aim of the present study was to explore the contribution of organic particle constituents and DEP to the pro-inflammatory effects of road tunnel PM. The AhR-inhibitor CH223191 and the anti-oxidant N-acetylcysteine (NAC) were used to assess the potential role of the AhR pathway and ROS-mediated mechanisms. Moreover, the contribution of DEP and mineral particles to the potency of road tunnel PM was assessed by comparing the responses to a sample of DEP and to particles derived from the stone material used in the asphalt of the respective tunnels.

## Results

### Pro-inflammatory responses and cytotoxicity to coarse, fine and ultrafine PM sampled in different road tunnels

The present study compares the pro-inflammatory responses to size-fractioned PM samples from the Marienborg and Hell road tunnels, both located in the Trondheim area of Norway. The particle samples have been characterized for the content of elemental carbon (EC) and OC, their ability to induce ROS formation in a cell-free system, content of endotoxin, and particle size distribution. The characteristics for the tunnel-derived particles have been previously published [29] and are also presented in an online supplementary table in the present study (Addition file 1 Table S1).

**Fig. 1:**
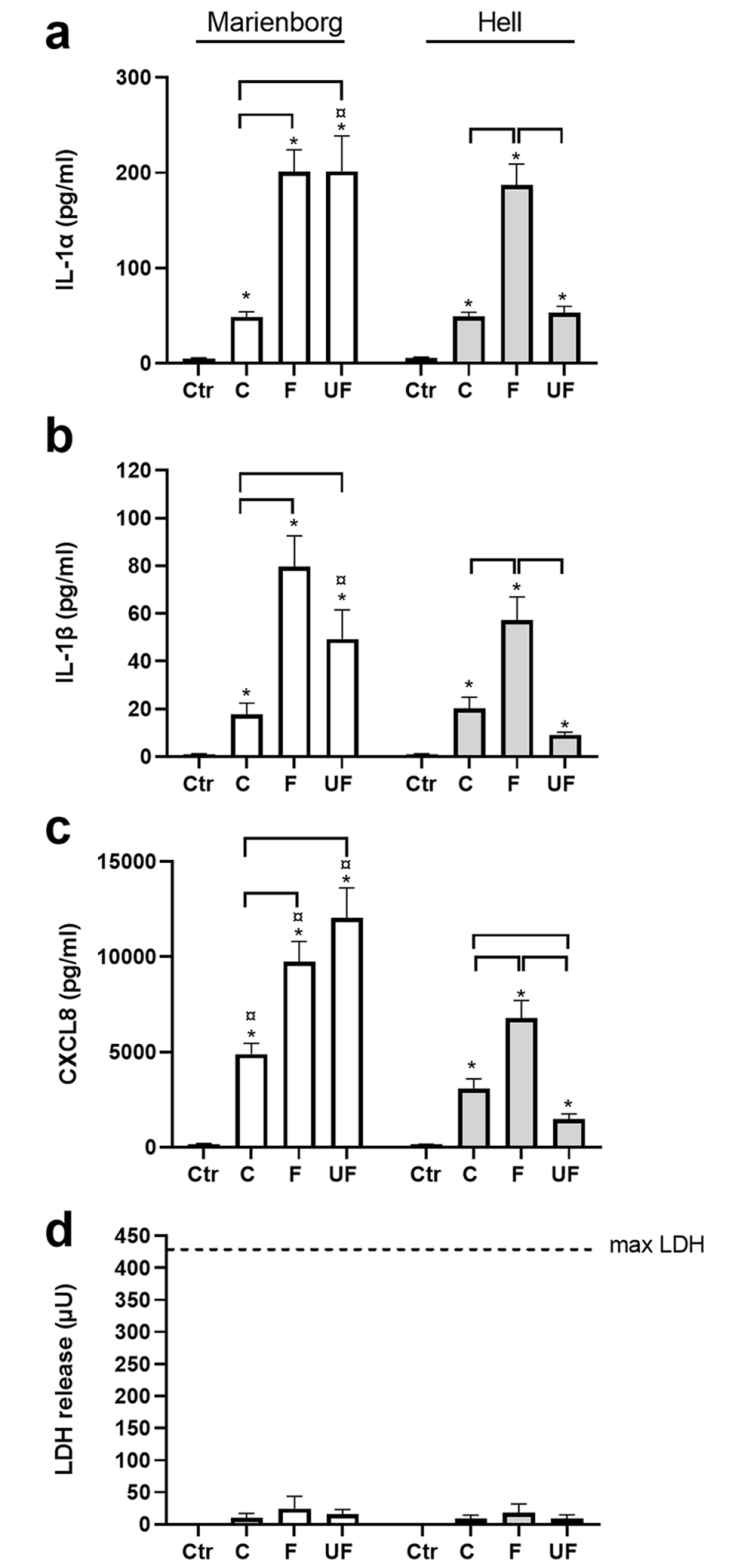
Cytokine release and cytotoxicity in HBEC3-KT cells after exposure to size-fractionated road tunnel PM. The cells were exposed to coarse (C: PM_10–2.5_), fine (F: PM_2.5–0.18_) and ultrafine (UF: PM_0.18_) PM sampled in the Marienborg (white bars) and Hell (grey bars) tunnels at a concentration of 100 µg/mL (10.4 µg/cm^2^) for 20 h. Release of IL-1α **(a)**, IL-1β **(b)**, CXCL8 **(c)** in the cell culture supernatant was analysed by ELISA, and cytotoxicity was measured by lactate dehydrogenase (LDH) release **(d)**. Data are presented as mean ± SEM of 3–19 independent experiments. *Significant increase compared to control. Brackets, significantly difference between different sizes of road tunnel PM. ^¤^Significantly different between tunnels at the same PM size. The statistical analysis was performed by 2-way ANOVA with Tukey’s multiple comparison test or Sidak’s multiple comparison test

The release of the pro-inflammatory cytokines interleukin-1α (IL-1α), IL-1β and the neutrophilic attractant CXC-motif chemokine ligand 8 (CXCL8/IL-8) [41, 42] was examined after exposure to 100 µg/mL (10.4 µg/cm^2^) coarse, fine and ultrafine PM sampled from the two road tunnels (Fig. 1). The fine and ultrafine fractions sampled in the Marienborg tunnel were the most potent and induced higher cytokine release than the coarse PM, whereas the fine fraction was the most potent in the Hell tunnel. When comparing the different size fractions from the two tunnels, all PM samples from the Marienborg tunnel induced higher CXCL8 release than the corresponding samples from the Hell tunnel. Moreover, ultrafine PM from the Marienborg tunnel induced higher levels of all cytokines than the corresponding sample from the Hell tunnel. None of the road tunnel samples increased the cytotoxicity in HBEC3-KT cells at the concentration of 100 µg/mL (10.4 µg/cm^2^), as measured by release of lactate dehydrogenase (LDH), after exposure for 20 h (Fig. 1). These results are in accordance with our previous study [29].

### PM-induced gene expression of pro-inflammatory cytokines and COX2

**Fig. 2:**
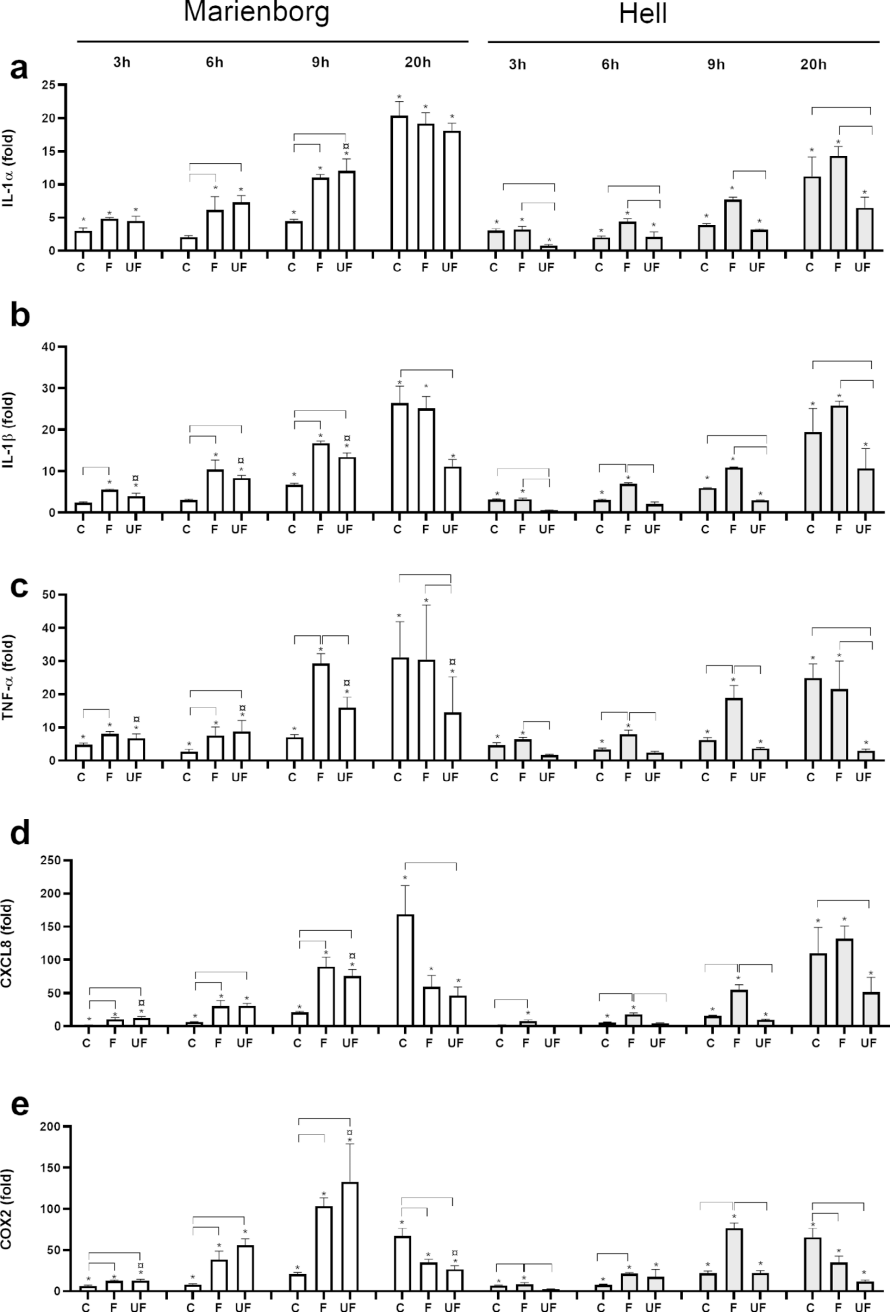
Time-dependent gene expression of IL-1α, IL-1β, TNF-α, CXCL8 and COX2 in HBEC3-KT cells after exposure to road tunnel PM. The cells were exposed to 100 µg/mL (10.4 µg/cm^2^) coarse PM (C), fine PM (F) and ultrafine PM (UF) from the Marienborg (white bars) and the Hell (grey bars) tunnels for 3, 6, 9 and 20 h. Gene expression of IL-1α **(a)**, IL-1β **(b)**, TNF-α **(c)**, CXCL8 **(d)** and COX2 **(e)** was analysed by qPCR. Results are presented as gene-expression (mean ± SEM) relative to the respective control (n = 4 independent experiments). *Significant increase compared to control at each time point. Brackets, significant differences between PM sizes. ^¤^Significantly different between tunnels at the same PM size and time-point. The statistical analysis was performed by 2-way ANOVA with Tukey’s multiple comparison test on normalized CT values

The gene expression of IL-1α, IL-1β, tumor necrosis factor-alpha (TNF-α), CXCL8, cycloxygenase-2 (PTGS2/COX2), was investigated from 3 to 20 h after exposure to coarse, fine and ultrafine PM from the two road tunnels (Fig. 2). Coarse, fine and ultrafine PM from both tunnels induced a time-dependent up-regulation of the expression of all genes after 3–6 h exposure (Fig. 2a-d). Fine PM tended to induce a higher increase in the expression of all these genes than coarse PM after 3–9 h exposure. However, after 20 h the IL-1α, IL-1β and TNF-α expression induced by the fine and coarse PM was approximately similar, while COX2 expression was even higher after exposure to coarse PM. Furthermore, the CXCL8 expression at 20 h was also higher for the coarse than the fine PM sampled in the Marienborg tunnel, but more similar in the Hell tunnel. Overall, this suggests a more delayed response to the coarse than fine and ultrafine PM samples. Ultrafine and fine PM from the Marienborg tunnel induced similar responses, with the exception of IL-1β and TNF-α expression at later time-points, which were higher after exposure to the fine PM sample. The potency of the ultrafine PM from Hell tunnel was lower than the corresponding fine PM in most cases. While the relative gene expression of all the cytokine genes induced by the coarse and fine PM from the two tunnels was similar, gene expression induced by the ultrafine PM from Hell was lower than the corresponding PM from the Marienborg tunnel, depending on time-point and gene (Fig. 2a-d).

### PM-induced gene expression of CYP1A1, CYP1B1 and PAI-2

**Fig. 3:**
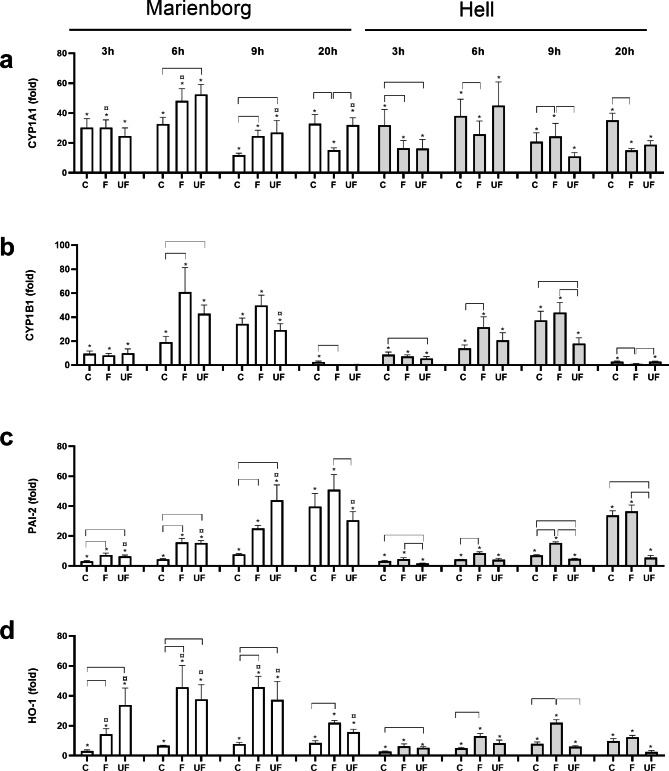
Time-dependent gene expression of CYP1A1, CYP1B1, PAI-2 and HO-1 in HBEC3-KT cells after exposure to road tunnel PM. The cells were exposed to 100 µg/mL (10.4 µg/cm^2^) coarse (C), fine (F) and ultrafine (UF) PM from the Marienborg (white bars) and Hell (grey bars) tunnels for 3, 6, 9 and 20 h. Gene expression of CYP1A1 **(a)**, CYP1B1 **(b)**, PAI-2 **(c)** and HO-1 **(d)** was analysed by qPCR. Results are presented as gene-expression (mean ± SEM) relative to the respective control (n = 4 independent experiments). *Significant increase compared to control at each time point. Brackets, significant differences between PM sizes. ^¤^Significantly different between tunnels at the same PM size and time-point. The statistical analysis was performed by 2-way ANOVA with Tukey’s multiple comparison test on normalized CT values

The time-dependent increases in the gene expression of cytochrome P450 (CYP)1A1, CYP1B1 and plasminogen activator inhibitor-2 (SerpinB2, PAI-2) were examined (Fig. 3). CYP1A1 and CYP1B1 are metabolic enzymes under transcriptional control of the AhR pathway that are involved in metabolic activation of organic chemicals like polycyclic aromatic hydrocarbons (PAH) [43, 44], while PAI-2 is an AhR-mediated enzyme involved in regulation of adaptive immunity [45]. Regarding CYP1A1, the coarse PM induced lower gene expression than the fine and ultrafine PM from Marienborg tunnel at 6 and 9 h, whereas the coarse PM was the most potent at 20 h. After exposure to the PM from the Hell tunnel, no consistent time-pattern was observed upon comparing the different size-fractions (Fig. 3a). When comparing the two tunnels, fine PM from the Marienborg tunnel induced significantly higher CYP1A1 expression at the early time-points than the corresponding sample from the Hell tunnel. Moreover, the ultrafine PM from the Marienborg tunnel induced a higher increase in expression of CYP1A1 than the ultrafine PM from the Hell tunnel at late time-points. No substantial difference in CYP1A1 expression could be observed for the coarse PM samples from the two tunnels (Fig. 3a). The time-dependent increase of CYP1B1-expression showed a peak at 6 to 9 h after exposure to PM samples from both tunnels. The different size fractions from the Marienborg tunnel showed no substantial differences in CYP1B1 expression. However, some minor differences were noted after 6 h exposure, where the fine and ultrafine PM samples induced significantly higher responses than the corresponding coarse PM samples. No consistent patters were observed when comparing the different size fractions from the Hell tunnel or between the corresponding size fractions of PM sampled in the two tunnels (Fig. 3b). The gene expression of PAI-2 peaked at 9 to 20 h after exposure to PM sampled in the Marienborg tunnel, and at 20 h after PM from the Hell tunnel (Fig. 3c). The fine and ultrafine PM sampled in the Marienborg tunnel induced higher gene expression than the coarse PM sample at early time-points, whereas no differences were observed after 20 h exposure. Regarding PM sampled in the Hell tunnel, the fine PM showed significantly higher PAI-2 expression at 6 and 9 h than the coarse PM fraction, but not at 20 h. The ultrafine PM from the Hell tunnel only induced low levels of PAI-2 expression. When comparing the responses to the PM sampled in the two tunnels, the ultrafine PM from Hell induced lower expression than the ultrafine PM from the Marienborg tunnel, while no significant differences were observed for the coarse and fine PM.

### PM-induced gene expression of heme oxygenase-1 (HO-1)

Heme oxygenase-1 (HMOX1/HO-1) is an antioxidant enzyme, often used as a marker of redox responses [46, 47]. Previously, we have shown the time-dependent relationship between ROS formation in bronchial epithelial cells after exposure to silica nanoparticle and subsequent induction of HO-1 at gene expression and protein levels [48]. As can be seen in Fig. 3d, all the size-fractionated particles from both tunnels induced significant increase of the gene expression for HO-1. The fine and ultrafine PM from the Marienborg tunnel induced early and marked increases in HO-1 expression that were higher than the expression after exposure to coarse PM. The differences between PM fractions sampled in the Hell tunnel were less marked. The fine PM showed higher gene expression than the coarse PM at 6 and 9 h. Moreover, the ultrafine PM from Hell tunnel tended to induce lower expression than the fine PM sample, but this was only significant at 9 h. When comparing the two tunnels, the HO-1 expression induced by fine and the ultrafine PM from the Hell tunnel was lower than for the effects from the corresponding PM samples from the Marienborg tunnel at most time-points, whereas the responses were more similar between the coarse PM samples (Fig. 3d).

### Comparison of the cytokine release of road tunnel PM, stone particles and DEP, and the role of AhR and ROS

**Fig. 4:**
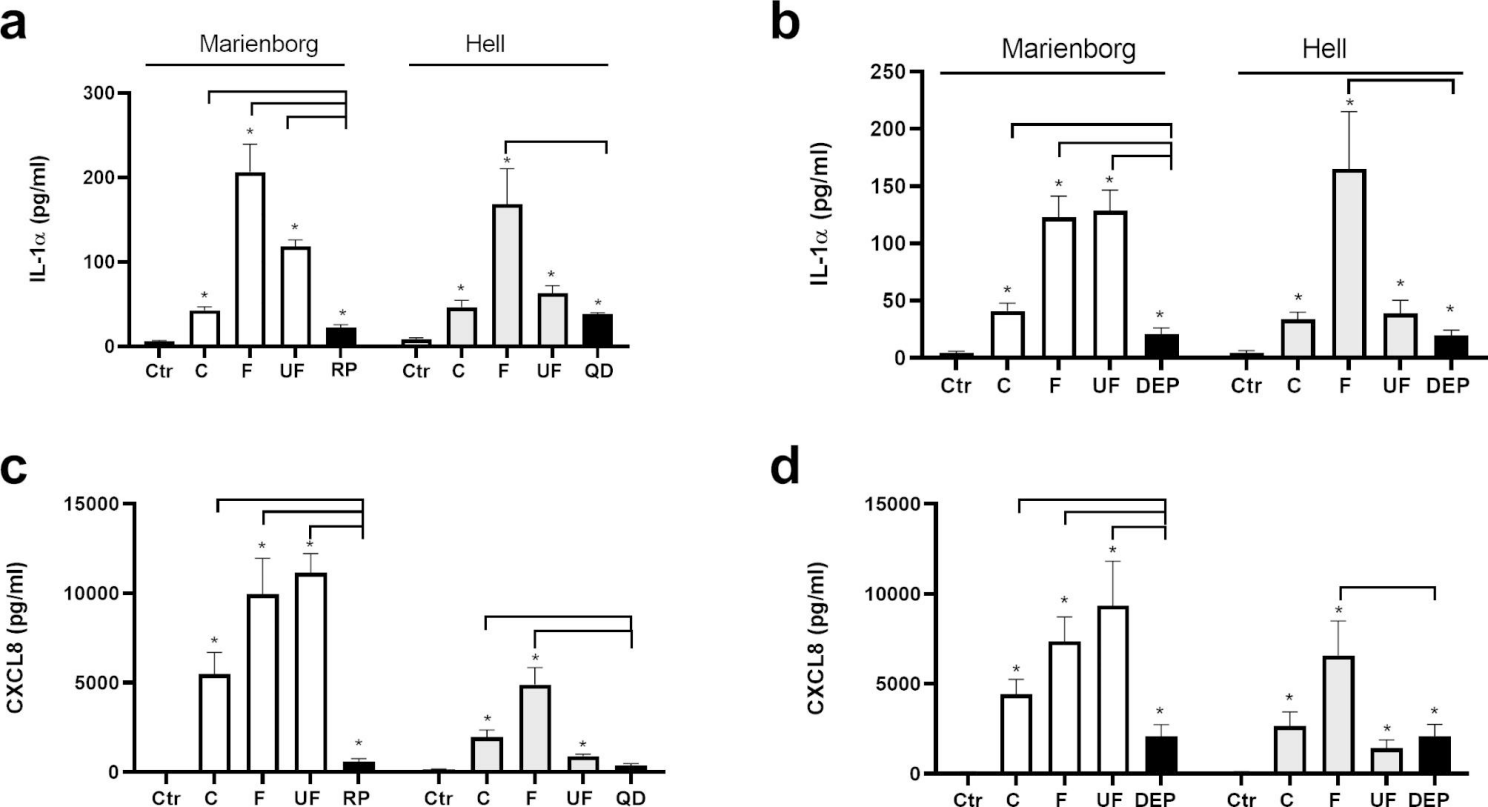
Comparison of road tunnel PM to stone particles and diesel exhaust particles (DEP) on cytokine release in HBEC3-KT cells. The cells were exposed to 100 µg/mL (10.4 µg/cm^2^) of coarse (C), fine (F) and ultrafine (UF) PM sampled in the Marienborg (white bars) and Hell (grey bars) tunnels for 20 h, and compared to the same concentrations (100 µg/mL (10.4 µg/cm^2^)) of particles from the stone materials rhomb porphyry (RP) and quartz diorite (QD) used in the respective tunnels (**a** and **c**, black bars) or to diesel exhaust particles (DEP) (**b** and **d**, black bars). Release of IL-1α **(a, b)** and CXCL8 **(c, d)** was analysed by ELISA. Results are presented as mean ± SEM of 7 independent experiments. *Significant different from control. Brackets, significantly different from the different sizes of road tunnel PM. The statistical analysis was performed by one-way ANOVA with Dunnett’s multiple comparison test

**Fig. 5:**
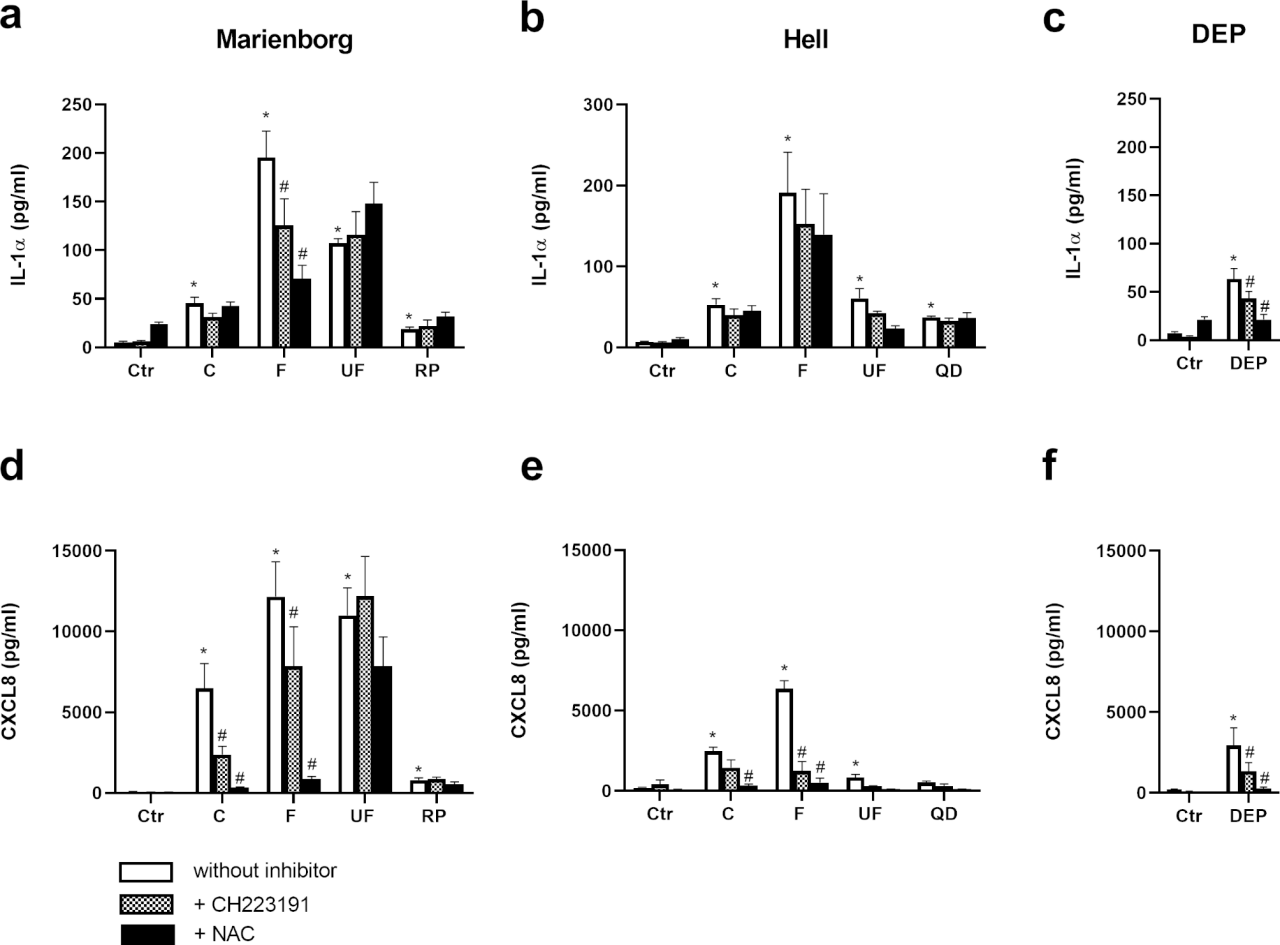
Role of AhR and ROS in cytokine release in HBEC3-KT cells after exposure to road tunnel PM particles, stone particles and DEP. The cells were exposed to 100 µg/mL (10.4 µg/cm^2^) of coarse (C), fine (F) and ultrafine (UF) PM sampled in the Marienborg and Hell tunnels and to 100 µg/mL (10.4 µg/cm^2^) particles derived from the stone materials rhomb porphyry (RP) and quartz diorite (QD) **(a, b, d, e)**. The cells were also exposed to 100 µg/mL (10.4 µg/cm^2^) DEP in separate experiments **(c, f)**. To assess the role of AhR and ROS, the cells were treated with the AhR-inhibitor CH223191 (1 µM) and the anti-oxidant N-acetyl cysteine (NAC, 5 mM) 1 h before and during particle exposure. Release of IL-1α **(a, b, c)** and CXCL8 **(d, e, f)** was determined by ELISA. The data presented represent the mean ± SEM of 4–5 independent experiments. *Significant different from control. ^#^Significant reduction compared to without inhibitor. The statistical analysis was performed by 2-way ANOVA with Dunnett’s comparison test

The pro-inflammatory response to road tunnel PM was compared with particles derived from the respective stone materials used in the pavement, and to DEP (Fig. 4). The HBEC3-KT cells were also treated with the AhR-inhibitor CH223191 and the anti-oxidant N-acetyl cysteine (NAC) to explore the role of AhR and ROS in PM-induced responses (Fig. 5). Exposure to rhomb porphyry particles induced lower IL-1α and CXCL8 release than the coarse, fine and ultrafine PM from the Marienborg tunnel (Fig. 4a, c). The differences between quartz diorite particles and the PM sampled in the Hell tunnel were of a lower magnitude. Quartz diorite showed lower responses than fine PM for IL-1α, and than coarse and fine PM for CXCL8 (Fig. 4a, c). Similarly, the responses to DEP, generated in an engine without a particle filter, containing relatively high levels of OC [49] were compared to the road tunnel PM. The DEP induced significantly lower IL-1α and CXCL8 release than observed for all PM size fractions from the Marienborg tunnel. In comparison, DEP only induced lower responses than the fine PM from the Hell tunnel (Fig. 4b, d).

*The AhR-inhibitor CH223191* reduced the IL-1α response for the fine PM from the Marienborg tunnel (Figur 5a), while no effects were seen on the responses after exposure to the other PM. However, the inhibitor reduced the release of CXCL8 following exposure both to coarse and fine tunnel PM from the Marienborg tunnel and fine PM from the Hell tunnel (Fig. 5d, e). Furthermore, the AhR-inhibitor significantly reduced IL-1α and CXCL8 responses to DEP (Fig. 5c, f), but had no effect on stone particle-induced cytokine release (Fig. 5a, b, d, e).

*The anti-oxidant NAC* caused only a signicant reduction in the IL-1α release induced by the fine Marienborg particles (Fig. 5a). In contrast, NAC markedly reduced the CXCL8 release induced by coarse and fine PM samples from both tunnels, while the responses induced by the ultrafine PM were not significantly affected (Fig. 5d, e). NAC significantly reduced the DEP-induced IL-1α and CXCL8 responses (Fig. 5c, f). For rhomb porphyry and quartz diorite no significant reductions were observed for any of cytokines after addition of NAC (Fig. 5a, b, d, e). The basal levels of IL-1α and CXCL8 were increased and slightly reduced, respectively, by NAC treatment (Fig. 5a, b, d, e).

### Comparison of pro-inflammatory cytokine gene expression of road tunnel PM to stone particles and to DEP, and the role of AhR and ROS

**Fig. 6:**
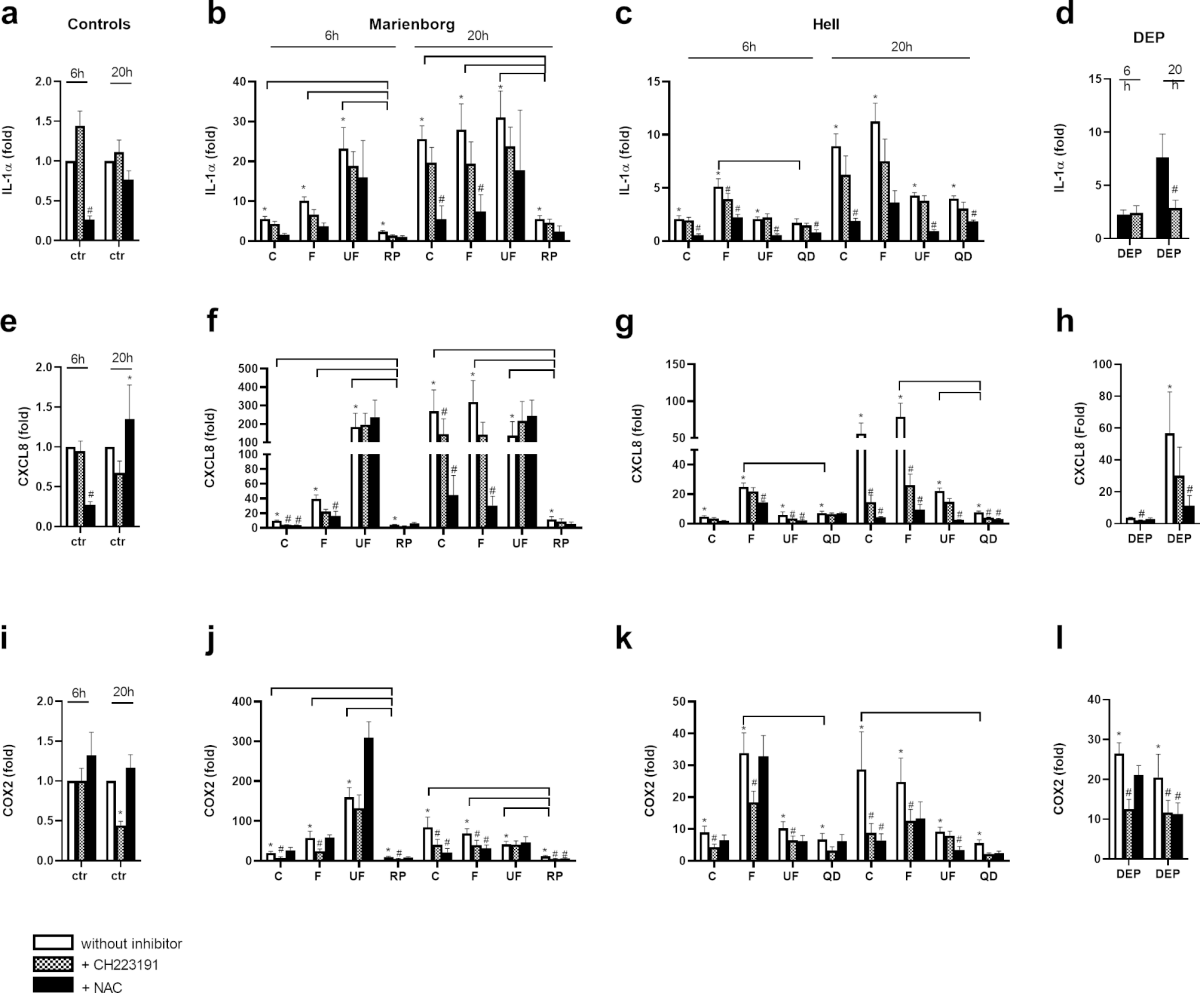
Comparison of road tunnel PM to particles from stone materials in the pavement and to DEP, and the role of AhR and ROS, in gene expression of IL-1α, CXCL8 and COX2 in HBEC3-KT cells. The cells were exposed to 100 µg/mL (10.4 µg/cm^2^) coarse (C), fine (F) and ultrafine (UF) PM sampled in the Marienborg and Hell tunnels and to particles derived from the stone materials rhomb porphyry (RP) and quartz diorite (QD) (100 µg/mL (10.4 µg/cm^2^)) **(b, c, f, g, j, k)**. The cells were also exposed to 100 µg/mL (10.4 µg/cm^2^) DEP in separate experiments **(d, h, l)**. To assess the role of AhR and ROS, the cells were treated with the AhR-inhibitor CH223191 (1 µM) and the anti-oxidant N-acetyl cysteine (NAC, 5 mM) 1 h before and during particle exposure. Gene expression of IL-1α **(a, b, c, d)**, CXCL8 **(e, f, g, h)** and COX2 **(i, j, k, l)** was measured after 6 or 20 h exposure by qPCR. The data are presented as the mean ± SEM of 4–5 independent experiments. *Significant different compared to control. Brackets, significant less responses of stone materials compared to the different sizes of road tunnel PM. # Significant reduction compared to cells without inhibitor. The statistical analysis was performed by two-Way ANOVA with Dunnett’s multiple comparison test

Differences between road tunnel PM, stone particles and DEP were also examined at the gene expression level. Rhomb porphyry particles induced lower expression of IL-1α, CXCL8 and COX2 than all the size fractions from the Marienborg tunnel after 6 and 20 h exposure (Fig. 6b, f, j). In comparison, the responses induced by the quartz diorite particles were more similar to PM sampled in the Hell tunnel. At 6 h, quartz diorite induced lower expression than the fine PM from the Hell tunnel for all genes (Fig. 6c, g, k). At 20 h, CXCL8 expression induced by quartz diorite was lower than for the fine and ultrafine PM, while for COX2 the expression was lower than for coarse PM (Fig. 6g, k). For DEP, marked inductions of the gene expression of IL-1α, CXCL8 and COX2 were observed. These effects on the gene expression level could not be directly compared to the responses to the road tunnel PM, as DEP was not examined in the same experiment as road tunnel PM (Fig. 6d, h, l).

*The AhR-inhibitor CH223191* gave small reductions of gene expression of IL-1α, with significant reduction only obtained for the fine Hell PM at 6 h (Fig. 6c). CH223191 reduced the gene expression of CXCL8 for the coarse PM from Marienborg tunnel at both 6 and 20 h, but not significantly for the fine PM. Furthermore, the AhR-inhibitor did not reduce the CXCL8 response to ultrafine PM. In comparison, reductions were observed for the ultrafine PM at 6 h, and coarse and fine PM at 20 h for PM from the Hell tunnel (Fig. 6f, g). Regarding COX2, the AhR-inhibitor reduced expression induced by coarse and fine PM from both tunnels at both time-points, whereas for the ultrafine PM significant reduction was only observed at 6 h from the Hell tunnel (Fig. 6j, k). The inhibitor also reduced the particle-induced responses to DEP, with reductions at both time-points for IL-1α and COX-2 expression and only at 6 h for CXCL8 (Fig. 6d, h, l). Furthermore, the inhibitor tended to reduce gene expression induced by the rhomb porphyry and quartz diorite particles, but the effects varied with the time-point and the gene in question.

*The anti-oxidant NAC* significantly reduced IL-1α expression induced by the coarse and fine PM from the Marienborg tunnel at 20 h and all the PM samples from Hell tunnel at 6 h (Fig. 6b, c). Regarding CXCL8, NAC reduced the gene expression induced by the coarse and fine PM from the Marienborg tunnel at both time-points, whereas the expression due to ultrafine PM exposure was not affected. NAC reduced the CXCL8 gene expression induced by the fine and ultrafine PM from the Hell tunnel at 6 h, and for the coarse, fine and ultrafine PM at 20 h (Fig. 6f, g). Regarding COX2, NAC did not affect the gene expression to neither coarse, fine nor ultrafine PM from the Marienborg tunnel at 6 h, whereas the expression induced by the coarse and fine PM was significantly reduced at 20 h. NAC did not affect the COX2 gene expression induced by any of the Hell tunnel PM samples at 6 h, but significantly reduced the expression for the coarse and ultrafine PM at 20 h (Fig. 6j, k). With respect to DEP, NAC reduced the gene expression of IL-1α, CXCL8 and COX2 at 20 h (Fig. 6d, h, l). NAC reduced the expression of these pro-inflammatory genes after exposure to the stone materials, but these effects were depending on the gene and time-points. For rhomb porphyry, the only significant reduction was observed for COX2 at 20 h (Fig. 6j). For quartz diorite, NAC significantly reduced the expression of IL-1α at 6 and 20 h, and CXCL8 at 20 h (Fig. 6c, g). The basal gene expressions of IL-1α and CXCL8 were only reduced after 6 h and not after 20 h, although the PM-induced expressions of the cytokines were markedly reduced, especially after 20 h.

### Comparison of gene expression related to xenobiotic and oxidative metabolism after exposure to road tunnel PM to stone particles and to DEP, and effect of AhR- and ROS-inhibition

**Fig. 7:**
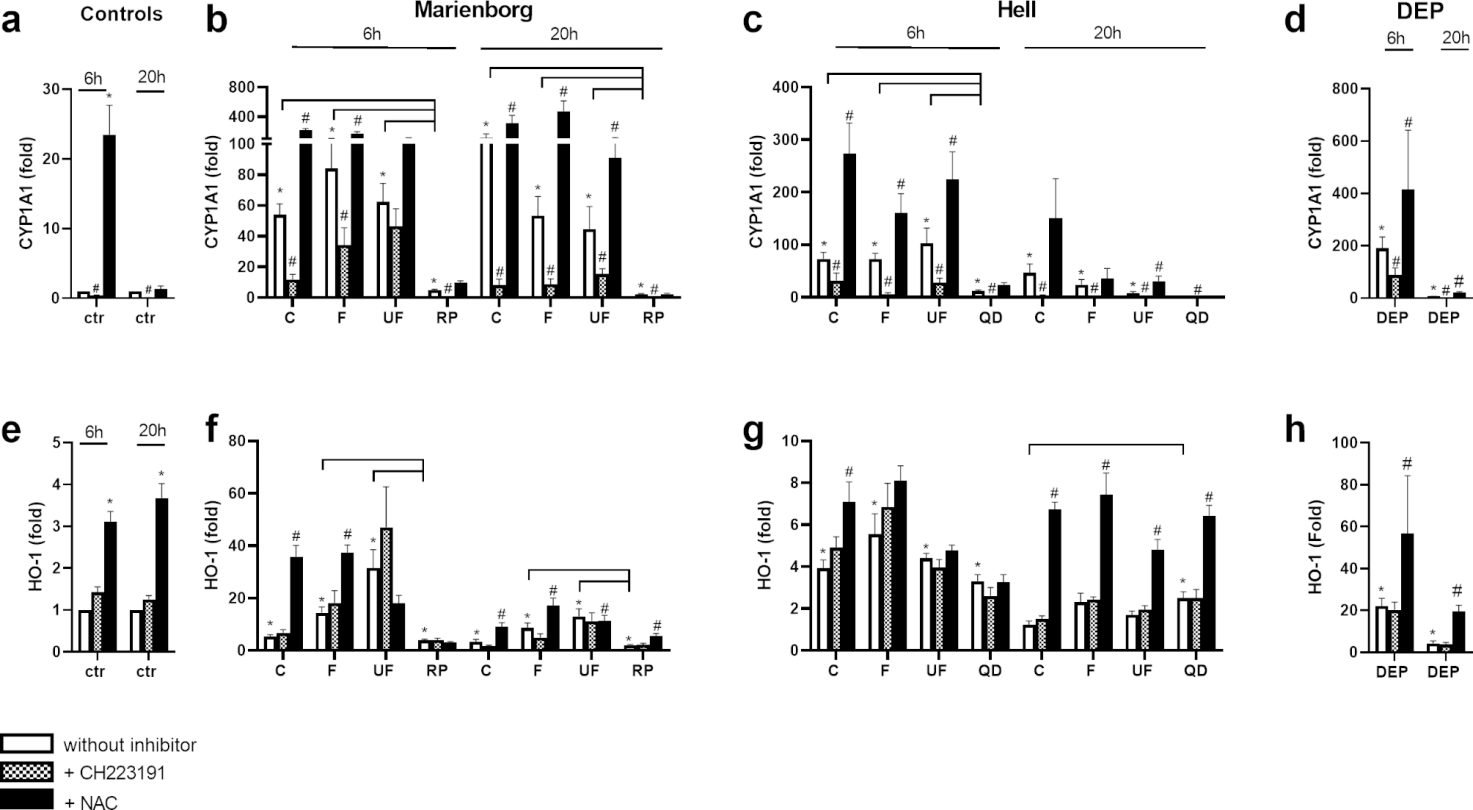
Comparison of road tunnel PM to particles from stone materials in the pavement and to DEP, and the role of AhR and ROS, in gene expression of CYP1A1 and heme oxygenase 1 (HO-1) in HBEC3-KT cells. The cells were exposed to 100 µg/mL (10.4 µg/cm^2^) coarse (C), fine (F) and ultrafine (UF) PM sampled in the Marienborg and Hell tunnels and to particles derived from the stone materials rhomb porphyry (RP) and quartz diorite (QD) (100 µg/mL (10.4 µg/cm^2^)) **(b, c, f, g)**. The cells were also exposed to 100 µg/mL (10.4 µg/cm^2^) DEP in separate experiments **(d, h)**. To assess the role of AhR and ROS, the cells were treated with the AhR-inhibitor CH223191 (1 µM) and the anti-oxidant N-acetyl cysteine (NAC, 5 mM) 1 h before and during particle exposure. Gene expression of CYP 1A1 **(a, b, c, d)** and HO-1 **(e, f, g, h)** was determined after 6 or 20 h exposure by qPCR. The data are presented as the mean ± SEM of 4–5 experiments. *Significant different compared to control. Brackets, significant less responses of stone materials compared to the different sizes of road tunnel PM. # Significant different when compared to cells without inhibitor. The statistical analysis was performed by two-Way ANOVA with Dunnett’s multiple comparison test

The CYP1A1 gene expression was higher after exposure to all size fractions of tunnel PM from Marienborg compared to rhomb porphyry particles after 6 and 20 h exposure. Similar results were observed when comparing the PM sampled in the Hell tunnel to quartz diorite particles, but the difference was only significant at 6 h (Fig. 7b, c). The induction of HO-1 expression after exposure to rhomb porphyry particles was also lower than for the fine and ultrafine PM from Marienborg at 6 and 20 h (Fig. 7f). In comparison, no significant differences in the expression of HO-1 were observed between the quartz diorite particles and the fine and ultrafine PM from the Hell tunnel (Fig. 7g). However, the quartz diorite particles induced a higher HO-1-response than the coarse PM after 20 h exposure (Fig. 7g). As for road tunnel PM, DEP induced marked expressions of CYP1A1 and HO-1, with most marked responses at 6 h (Fig. 7d, h).

*The AhR-inhibitor CH223191* markedly reduced the gene expression of CYP1A1 induced by all the fractionated size samples of PM from both tunnels at both 6 and 20 h, with the exception of the ultrafine PM from the Marienborg tunnel at 6 h (Fig. 7b, c). The DEP-induced increase in CYP1A1 was reduced at both time-points (Fig. 7d). Similarly, the inhibitor also reduced the small increase in CYP1A1 expression induced by rhomb porphyry and quartz diorite particles at 6 and 20 h (Fig. 7b, c). Particle-induced expression of HO-1 was not affected by the the AhR-inhibitor (Fig. 7f, g, h).

*The anti-oxidant NAC* markedly augmented the CYP1A1 gene expression induced by the size-fractionated samples of PM from both tunnels at 6 and 20 h, with the exception of the ultrafine PM from the Marienborg tunnel at 6 h and coarse and fine PM from the Hell tunnel at 20 h (Fig. 7b, c). A similar increase was observed for NAC on the expression of CYP1A1 after exposure to DEP (Fig. 7d). Treatment with NAC did not increase the effect of the stone materials on expression of CYP1A (Fig. 7b, c). NAC treatment also augmented the HO-1 expression after treatment with coarse and fine PM from the Marienborg tunnel at both time-points. With respect to the PM sampled in the Hell tunnel, NAC increased the HO-1 induction by all the size-fractionated samples at 20 h, whereas only the effect of coarse PM was increased significantly at 6 h (Fig. 7f, g). NAC also increased the effect of DEP at both time-points (Fig. 7h). The induction of HO-1 by rhomb porphyry and quartz diorite was also enhanced by treatment with NAC after 20 h exposure (Fig. 7f, g).

## Discussion

The main aim of the present study was to examine to which extent the pro-inflammatory responses of road tunnel PM could be attributed to combustion-derived DEP and wear particles from the road pavement, and whether the responses could be attributed to AhR- and ROS-mediated mechanisms. Following exposure to coarse, fine and ultrafine road tunnel PM gene expression and release of the pro-inflammatory cytokines IL-1α, IL-1β and CXCL8 in HBEC3-KT cells were markedly increased. Increased gene expression of COX2, CYP1A1, CYP1B1, PAI-2 and HO-1 was also observed. On a similar mass basis, DEP exposure induced lower cytokine expression and release than all PM samples from the Marienborg tunnel and lower cytokine expression and release than fine PM from the Hell tunnel. The expression of CYP1A1 showed qualitative similarities between DEP and road tunnel PM. Furthermore, particles derived from the stone material rhomb porhyry induced lower cytokine gene expression and release than all PM fractions from the Marienborg tunnel, whereas quartz diorite particles induced lower cytokine gene expression and release than coarse and/or fine PM from the Hell tunnel, depending on the cytokine. The AhR-inhibitor CH223191 reduced release and expression of IL-1α and CXCL8, and expression of COX2, for fine and coarse PM, depending on the particle sample and time-point, while no substantial effects were observed for ultrafine PM from either tunnel. Whereas particle-induced expression of CYP1A1 was markedly reduced by CH223191, HO-1 expression was not affected. Moreover, the anti-oxidant NAC reduced the release and expression of IL-1α and CXCL8, and COX2-expression, but augmented the expression of CYP1A1 and HO-1 to the coarse and fine PM. As observed for the AhR-inhibitor CH223191 the cytokine responses to ultrafine PM were not affected by NAC.

### Comparison of different PM size fractions on the gene expression levels

The present study confirms and extends our previous findings on road tunnel PM-induced cytokine release [29]. Here we report that the gene expression of IL-1α, IL-1β, TNFα and CXCL8 was up-regulated from 3 to 20 h, indicating that the particle-induced cytokine release was associated with increased transcription of the respective genes. In addition, up-regulation of COX2, CYP1A1, CYP1B1, PAI-2, and HO-1 was detected after exposure to the road tunnel PM. The responses to coarse PM were less pronounced than for fine PM at the early time points, in accordance with the lower release of pro-inflammatory cytokines. The higher inflammatory responses of fine versus coarse traffic-related PM in epithelial lung cells is consistent with findings reported in a few other studies [29–31]. However, the relative pro-inflammatory potential of coarse versus fine PM may vary with experimental model, as coarse PM has been observed to be the most potent in macrophages [19–26], and in animal models [19]. In the present study, it is interesting to note that while coarse PM tended to induce lower responses at early time points, the expression of several of the genes measured were equal or higher to fine and ultrafine PM after 20 h exposure, suggesting that different mechanisms are important for the different size fractions. Furthermore, the magnitude of cytokine release and expression induced by the ultrafine PM differed between the two tunnels. While ultrafine PM sampled in the Marienborg tunnel induced responses of a similar magnitude as the fine PM, these responses were lower for the ultrafine PM than the fine PM sampled in the Hell tunnel. This is likely stemming from differences in the composition of the ultrafine PM from the two tunnels, presumably due to differences in temperature, traffic load, and number of heavy vehicles between the tunnels.

### The contribution of stone materials, DEP and OC to the effects of road tunnel PM

PM from traffic consists of a mixture of particles from fuel combustion and from wear of the road surface, tires and brakes [50, 51] which may vary in potency [52, 53]. The present study shows that road tunnel PM induced stronger pro-inflammatory responses than particles derived from the rhomb porphyry and quartz diorite stone materials used in the asphalt of the tunnels. In line with our previous findings on cytokine release [29], the road tunnel PM strongly up-regulated the expression CXCL8, IL-1α, COX2, CYP1A1, and HO-1, while only a small or no increase in the expression of these genes was induced by the stone materials, although the relative differences varied between endpoints and the two tunnels. Thus, the results suggest that the pro-inflammatory potency of road tunnel PM cannot be attributed to the stone materials alone.

In our previous study, we found that the pro-inflammatory responses of coarse, fine and ultrafine PM from six different samples of road tunnel PM were associated with the content of organic carbon (OC) content [29], suggesting that OC could be important for triggering the responses. The percentages of OC in the road tunnel PM used in the present study were rather high, constituting approximately 50% of the mass for the ultrafine and fine PM, and 10% in the coarse fraction [29], see also Additional file 1 Table S1A). Interestingly, the coarse PM sample also induced CYP1A1 expression with almost equal levels as the fine PM at late time-points, despite the lower levels of OC in this sample. The OC in the tunnel PM samples can potentially originate from both fuel combustion (diesel and gasoline exhaust) and from non-combustion sources (bitumen, tires). The relative amount of OC and the specific chemical composition of DEP is highly dependent on the fuel source, as well as the engine-, filter- and catalysator technology [49]. While the release of primary particles from modern diesel cars is rather minimal due to after-treatment of exhaust with particle filters, the effects on OC-emissions remains less clear [54, 55]. However, the high percentage of heavy duty vehicles in the Marienborg and Hell tunnels may suggest that diesel exhaust emissions may contribute significantly to the high levels of OC. The release of OC from gasoline cars may also be an important source, and it is known that the gas phase of exhaust from gasoline vehicles contains high levels of OC that are converted to secondary aerosols [54, 56].

DEP has been reported to induce pro-inflammatory responses in animal- and cell culture studies [10, 11], and several studies report that organic chemicals are important for the pro-inflammatory effects of DEP [11, 57–59]. Notably, the DEP used in the present study induced markedly lower cytokine responses than all the PM size fractions sampled in the Marienborg tunnel, and lower responses compared to the fine PM sampled in the Hell tunnel, suggesting that the pro-inflammatory potency of road tunnel PM cannot be attributed to primary DEP emissions alone. Moreover, the effects of the particle samples were compared on an equal mass basis, thus the relative differences between the tunnel PM and DEP would have even been greater if the concentrations had been adjusted for the amount of DEP in the PM.

In addition to fuel emissions, OC may also originate from non-combustion sources, such as bitumen in the asphalt or from tires [60, 61]. In support of bitumen or tyre wear particles as a potential contributors to the responses induced by traffic-derived PM, a study in human monocyte-derived macrophages reported that particles generated in a road wear simulator, consisting of a mixture of mineral particles, bitumen and tyre wear particles, induced pro-inflammatory responses of the same magnitude as PM sampled from a street [62]. Interestingly, the study also showed that particles from asphalt containing granite were more potent than particles from asphalt containing quartzite, indicating that the potency of road wear particles also may depend on the type of stone material [62]. However, a follow-up study in another model system (RAW264.7 macrophages), with the same particles from the road simulator showed substantially lower responses than street PM [52] similar to the observations in the present study.

Considering that traffic-derived PM consists of a mixture of particles from different sources, the effects are presumably due to the combined interactions of multiple PM constituents. We have recently reported that mineral particles, including the rhomb porphyry and quartz diorite samples used in the present study, increased the pro-inflammatory effects of DEP in HBEC3-KT cells when administered in combination [11]. Thus, the contribution of a type of particle to a complex PM mixture may potentially be greater than indicated by studies of the particle type separately. It should also be considered that other types of particles present in traffic-derived PM shown to induce inflammation in experimental studies, such as tire wear or brake wear particles [53, 63], could potentially induce similar effects in combination with DEP or minerals, which may contribute to the magnitude of the responses.

### Role of of AhR- and ROS-mediated mechanisms in the pro-inflammatory responses induced by road tunnel PM

We have previously reported that the pro-inflammatory responses induced by size-fractionated PM sampled in the two tunnels correlated significantly with the OC content of the different particle samples. This correlation was most pronounced for the most volatile OC fraction (evaporated at 310 °C), which likely also contains the majority of polycyclic aromatic hydrocarbons (PAH) [29]. As the activation of AhR is known to be important for the cellular responses to organic components [37, 43, 44], we examined the effects of the AhR-inhibitor CH223191 on the particle-induced pro-inflammatory responses. CH223191 has been found to reduce CYP expression and pro-inflammatory responses following exposure to PAH [64]. PAH are considered to be important constituents in OC extracts from DEP [65], and AhR-mediated pathways have been found to be involved in pro-inflammatory responses [11, 34, 37]. In the present study, CH223191 reduced the expression and release of CXCL8 induced by coarse and fine road tunnel PM and DEP to approximately the same magnitude, which may support that PAH and/or other hydrocarbons acting via AhR are important PM determinants for the pro-inflammatory responses. Overall, this supports the association between OC-content and pro-inflammatory responses in HBEC3-KT cells observed in our previous study of road tunnel PM [29].

The mechanisms for the AhR-mediated PM-induced responses are complex. The classical AhR-ARNT-signalling pathway regulates expression of xenobiotic metabolizing enzymes such as CYP1A1 and CYP1B1 [36, 43]. However, the AhR-Arnt signalling also regulates a number of pro-inflammatory cytokines directly through xenobiotic response elements in their promotor regions [66, 67]. In addition, AhR may also dimerize with various other transcription factors, including NF-kB subunits, such as RelB, or activate intracellular Ca^2+^-signalling and the EGFR-ERK1/2 pathway which both may affect expression of pro-inflammatory genes [44, 68–70]. Notably, AhR-ARNT-signaling regulates CYP1 enzymes that are central to metabolize PAH to reactive electrophilic metabolites with simultaneous formation of ROS, which may contribute to induction of inflammatory responses [32]. Such mechanisms have been suggested to be involved in development of airway diseases upon exposure to traffic-related PM [44]. However, in the present study treatment with NAC markedly augmented the gene expression of CYP1A1 and HO-1 induced by the coarse and fine road tunnel PM and DEP. The mechanisms underlying this observation remain unclear, but as the effect was observed in both particle-exposed cells and unexposed controls, NAC likely interferes with some basal permissive response affecting both CYP1A1 and HO-1 expression. As recently reviewed elsewhere, the effects of NAC on redox regulation are complex and context-dependent, but the mechanisms are still not understood [71]. Interestingly, NAC has been reported to induce activation of Nrf2 [71]. Nrf2 regulates HO-1, but there is also considerable cross-talk between Nrf2 and AhR signalling, and Nrf2 may induce expression of both AhR and CYP1A1 [72]. This could explain the joint augmentation of HO-1 and CYP1A1 by NAC. More importantly, the AhR-inhibitor CH223191 suppressed baseline and PM-induced CYP1A1 expression, but did not reduce the up-regulation of HO-1. Although ROS production was not measured in the present study, this suggests that the HO-1 response cannot be attributed to induction of CYP1A1-mediated metabolism of PAHs, but likely derives from other redox-regulated pathways. While the role of ROS and redox signalling remains to be clarified, the effects of CH223191 and NAC provide some clues on the involvement of AhR. Both CH223191 and NAC inhibited the coarse and fine PM-induced pro-inflammatory responses despite the opposing effects on CYP1A1 expression. Based on this it seems likely that the role of AhR in mediating the pro-inflammatory responses of tunnel PM was not linked to activation of the classical AhR-Arnt pathway and activation of CYP1A1, but rather due to non-classical genomic or non-genomic signalling.

In contrast to coarse and fine PM, the pro-inflammatory responses to ultrafine PM were not significantly reduced by the AhR- and the ROS-inhibitors. This was observed even if the levels of OC were nearly as high for the ultrafine PM as for the fine PM in both tunnels. The lack of an AhR-mediated response, despite the high content of OC and PAH in these particle samples, could be explained by higher content of antagonistic AhR ligands [37]. This may suggest that the organic constituents in ultrafine PM are different from the fine road tunnel PM, and act via AhR-independent mechanisms. In line with this, previous studies have shown that pro-inflammatory responses in lung epithelial cells exposed to DEP or DEP-associated compounds may also be mediated by other mechanisms than AhR, such as activation of epidermal growth factor receptor (EGFR), G-protein-coupled receptors (GPCRs) and Ca^2+^ signalling [73–75]. The lack of AhR-inhibition on the pro-inflammatory responses of the ultrafine PM samples, might also reflect that other PM components than OC are important for the pro-inflammatory outcome of the ultrafine PM, differently from the fine and coarse road tunnel PM. Previously it has been reported that ultrafine PM sampled near a heavily trafficked road contained metals potentially associated with adverse health effects [76].

## Concluding remarks

Traffic-derived PM was sampled in two road tunnels in Norway, and the coarse, fine and ultrafine size fractions caused a time-dependent increase in the expression of pro-inflammatory cytokines, and of genes linked to oxidative and xenobiotic metabolism in HBEC3-KT cells. On an equal mass basis particles derived from the stone materials used in the road pavement induced lower cytokine expression and release than the corresponding road tunnel PM, although this varied with the tunnel, size fraction, response and time-point, and was most prominent compared to the fine PM samples. Similarly, the cytokine release induced by DEP was lower in comparison to the responses of the size-fractionated road tunnel PM. The pro-inflammatory responses to the coarse and fine fractions of road tunnel PM, but not the ultrafine, were partly reduced by the AhR-inhibitor CH223191, suggesting a role for AhR-dependent mechanisms for the largest fractions. The anti-oxidant NAC also reduced the particle-induced pro-inflammatory responses after exposure to fine and coarse tunnel PM and to DEP, indicating a role for ROS in the cellular signalling pathway(s). The lack of effect of the AhR-inhibitor on particle-induced HO-1 expression suggests that the ROS-mediated effects are independent of AhR. Furthermore, the role of AhR in mediating the pro-inflammatory responses of tunnel PM seems not linked to activation of the classical AhR-Arnt pathway and activation of CYP1A1. Overall, the observed role of AhR-mediated mechanisms may confirm a role for OC in the pro-inflammatory responses for the road tunnel PM, although other PM components and mechanisms also seem to contribute, especially with regard to the ultrafine particles. DEP is suggested to be involved in the OC-linked part of the PM-induced pro-inflammatory responses, even if other OC-sources could also be of importance. Future studies should address how the road tunnel affect the pro-inflammatory responses in other cell types, like macrophages.

## Materials and methods

### Materials

The cell culture medium LHC-9 and DMEM:F12, and Trypsin-EDTA, were bought from Gibco, Thermo Fisher Scientific, Waltham, MA USA. The cell culture flasks were obtained from Nunc A/S, Roskilde, Denmark, while the cell culture plates were from Corning, NY 14,831 USA. PureCol^™^ collagen was from Advanced BioMatrix, Inc, CA, USA.The ELISA cytoset for CXCL8 was purchased from Invitrogen, Thermo Fisher Scientific, Waltham, MA, USA, while IL-1α and IL-1β DuoSet were from R&D Systems, Inc, Minneapolis, MN, USA. Cytotoxicity Detection Kit (lactate dehydrogenase; LDH) was obtained from Merck KGaA, Darmstadt, Germany. The RNA isolating kit; NukleoSpin RNA plus, was bought from MACHEREY-NAGEL, Duren, Germany. CH22319 and N-acetyl cystein (NAC) were bought from Merck KGaA, Darmstadt, Germany. High Capacity cDNA Archive Kit, TaqMan Universal PCR Mastermix, TaqMan Gene Expression Assays (CXCL8, Hs00174103_m1, IL-1α; Hs00174092_m1, IL-1β; Hs01555410_m1, TNF-α; Hs01113624_g1, HO-1; Hs01110250_m1, CYP1A1; Hs01054797_g1, CYP1B1; Hs02382916_s1, PAI-2 ; Hs01010736_m1, COX2; Hs00153133_m1 and GAPDH; Hs02758991_g1) were bought from Applied Biosystems, Thermo Fisher Scientific, Waltham, MA, USA. Other chemicals were purchased from commercial sources at the highest purity available.

### Particle sampling and characterization

***Road tunnel PM.*** Traffic-derived coarse particles (PM_10-2.5_), fine particles (herein defined as PM_2.5-0.18_) and ultrafine PM (herein defined as PM_0.18_) were collected in two road tunnels in Trondheim, Norway, upon dry and humid road surface conditions and before and after road cleaning. The two road tunnels, Marienborg and Hell tunnel, were paved with asphalt containing the stone types rhomb porphyry and quartz diorite, respectively [29]. In the present study, PM from both tunnels sampled during humid road surface conditions was selected for in depth studies. Visual status of the road surface was used to classify the road surface as humid. The tunnel characteristics, sampling procedure and characterization of the road tunnel PM have been described previously [29]. Briefly, tunnel characteristics for the Hell tunnel were; average tunnel temperature 14.9 °C, relative humidity 60.5%, speed limit 80 km/h, approximately 16,000 vehicles each 24 h, 40% with studded tires and 17% heavy vehicles. For the Marienborg tunnel the average temperature was 7.3 °C, relative humidity 83.2.%, speed limit 60 km/h, 7000 vehicles each 24 h, 25% with studded tires and 10% heavy vehicles. The different PM size fractions were sampled 10 days after a comprehensive washing of the tunnel, using a high-volume cascade impactor (HVCI) with a multistage round slit nozzle at a flow rate of 900 L/h in 10–12 h periods. All samplings in each tunnel were performed from Sunday night until Thursday afternoon. Polyurethane foam (PUF) was used as impaction substrate for the coarse and fine PM, and a Whatman TE 38 filter for the ultrafine PM. The collected PM samples were extracted from the PUFs and filters by methanol, and vortexed/sonicated to dislodge the particles from the filters. The methanol was removed by a rotary evaporator. The PM samples were suspended in pyrogen-free sterile water (10 mg/mL), vortexed, sonicated and stored at − 20 °C. Before use the PM was vortexed and sonicated in a water bath for 60 s. The particles were characterized for content of total carbon, elementary carbon (EC) and organic carbon (OC), the ratio of OC to total PM mass, endotoxin content, and ability to generate acellular ROS (Additional file 1 Table S1a, see also Skuland et al.2022 [29]. The hydrodynamic size distributions of the PM samples are shown in Additional file 1 Table S1b; see also Skuland et al.2022 [29].

***Stone materials.*** The generation and characterization of the stone particle samples has been described in a previous study [40]. Briefly, stone aggregate samples of the stone materials were ground in a Los Angeles test machine and the resulting fine-grained materials were sieved to collect the portion of particles < 63 μm. Particle sizes < 10 μm were separated by gravitational settling in deionized water at room temperature for a duration determined by solving the stokes equation for time. All < 10 μm particles were centrifuged for 65 min at 9500 RPM in a Beckman Coulter Avanti J-26 XP centrifuge, and the particle samples were dried in a freeze–drier. The two stone materials were also prepared in pyrogen-free water in the same manner as the road tunnel PM, and stored at -20 °C. Just before use, the stone materials were sonicated in a water bath for 60 s, and vortexed before pipetting into culture wells. The stone particle samples have been characterized for mineral composition, metals and endotoxin content [40].

***Diesel exhaust particles (DEP).*** The DEP was collected from an engine without a diesel particle filter, such filter is necessary to fulfil EURO 5 requirements. DEP was dissolved in pyrogen-free water and stored at -20 °C [49]. Just before use, DEP was sonicated in a water bath for 60 s, and vortexed before pipetting into culture wells. The DEP has been characterized with respect to organic and metal constituents [49].

### Cell culture and exposure conditions

HBEC3-KT cells (passage 4–35; (ATCC CRL-4051) were maintained in LHC-9 medium in collagen-coated flasks (PureColTM) in a humidified atmosphere at 37 °C with 5% CO_2_, and with refreshment of medium every second day. For experiments the cells were seeded on pre-coated 6-well plates (170.000 cells/cm^2^) three days before exposure. The culture medium was changed to serum-free DMEM:F12 one day before exposure. The cells were exposed to 100 µg/mL (10.4 µg/cm^2^) of the different particle samples for 3–20 h, depending on the endpoint measured. This dose was based on previous experiments and may be in the range relevant for high urban exposure in the tracheobronchial region of the airways [77]. In selected experiments, the cells were pre-treated for 1 h prior and during PM exposure with an inhibitor of AhR, CH223191 (1 µM), and the anti-oxidant NAC (5 mM).

### Measurement of cell viability

To examine cell viability, LDH-release was measured in the cell culture media after 20 h of exposure and compared to maximal release (cells exposed to 2% Triton X-100 in media for 10 min). The measurements and calculations were done according to the manufacturer’s instructions (Roche, Germany).

### Cytokine analysis

Cell culture medium was collected and centrifuged at 300 x g to remove cell debris and at 8000 x g to remove floating particles. CXCL8, IL-1α and IL-1β protein levels were determined by sandwich ELISA according to the manufacturer’s guidelines. Absorbance was measured and quantified by a plate reader (TECAN Sunrise) equipped with a dedicated software (Magellan V 1.10).

### Gene expression analysis

RNA was isolated from cells using the NukleoSpin RNA plus according to the supplier’s recommendations. For these analyses 0.5-1 µg total RNA was reverse transcribed to cDNA in 25 µL (using a High-Capacity cDNA Archive Kit). After the cDNA synthesis, the cDNAs were diluted 1:100 in a solution of nuclease-free water, TaqMan Universal master mix and TaqMan Gene Expression. The expression of GAPDH, CXCL8, IL-1α, IL-1β, TNF-α, CYP1A1, CYP1B1, COX2, PAI-2 and HO-1 was analysed by qPCR using BioRads CFX96 Touch Real-Time PCR Detection System, with pre-designed TaqMan Gene Expression Assays and TaqMan Universal PCR Master Mix. The expression of each gene of interest in each sample was normalized against GAPDH and expressed as fold change compared to the untreated control as calculated by the ΔΔCt-method [78].

### Statistical analyses

The statistical analyses were performed by using GraphPad Prism software (version 9.0 Inc., San Diego, CA). The cytokine data were transformed to ensure normality, and then we used 2-way ANOVA with repeated measurements (except for the data in Fig. 4 were we have used one-way ANOVA with repeated measurments). Geisser-Greenhouse correction was used to account for non-sphericity of the data. Tukey’s multiple comparison test was used to compare data inside the groups (Marienborg or Hell tunnel) or Sidak’s multiple comparison test between groups (Marienborg vs. Hell tunnel). Dunnett’s comparison test was used as a post test to analyse the effect of inhibitors. To investigate the difference between stone materials and DEP versus road PM, one-way ANOVA with Dunnett’s multiple comparison test was used (Fig. 4). For the gene expression data we used the normalized ΔCt-values and performed 2-way ANOVA with Tukey’s multiple comparison test with repeated measurements.

### Electronic supplementary material

Below is the link to the electronic supplementary material.

Additional File 1: Table S1: PM characterization of coarse, fine and ultrafine PM sampled in two different road tunnels (Marienborg, and Hell) under humid road surface conditions; **a**) Total- and organic carbon (OC), endotoxin content and oxidative potentials (OP^DTT^) **b)** Hydrodynamic size distributions are shown as PM_10 − 2.5_, PM_2.5−0.1_ and PM_0.1_, measured in each of the sampled size fractions (coarse, fine and ultrafine PM) from the two tunnels. This was based on an equal area under curve (AUC) for the mass of the three fractions (in percentage of total). The PM_1_ values are also presented

## Data Availability

The datasets used in the presents study are available from the corresponding author upon reasonable request.

## Abbreviations

AhR: Aryl hydrocarbon receptor activities
CXCL8: Chemokine CXC-motif ligand 8
COX2: Cyclooxygenase 2
CYP: Cytochrome P450
DEP: Diesel exhaust particles
HO-1: Heme oxygenase-1
IL: Interleukin
LDH: Lactate dehydrogenase
NAC: N-acetyl salicylic acid
OC: Organic carbon
PM: Particulate matter
PAI-2: Plasminogen-activated inhibitor 2
PAH: Polycyclic aromatic hydrocarbons
PM: Particulate matter
TNF-α: Tumor necrosis factor-α
ROS: Reactive oxygen species
SEM: Standard error of mean
